# Synergy between NIR luminescence and thermal emission toward highly sensitive NIR operating emissive thermometry

**DOI:** 10.1038/s41598-020-76851-3

**Published:** 2020-11-12

**Authors:** Lukasz Marciniak, Karolina Trejgis, Radosław Lisiecki, Artur Bednarkiewicz

**Affiliations:** Włodzimierz Trzebiatowski Institute of Low Temperature and Structure Research, Wrocław, Poland

**Keywords:** Chemical engineering, Optical materials and structures

## Abstract

There are many figures of merit, which determine suitability of luminescent thermometers for practical applications. These include thermal sensitivity, thermal accuracy as well as ease and cost effectivness of technical implementation. A novel contactless emission thermometer is proposed, which takes advantage of the coexistence of photoluminescence from Nd^3+^ doping ions and black body emission in transparent Nd^3+^ doped-oxyfluorotellurite glass host matrix. The opposite temperature dependent emission from these two phenomena, enables to achieve exceptionally high relative sensitivity S_R_ = 8.2%/°C at 220 °C. This enables to develop new type of emissive noncontact temperature sensors.

## Introduction

**Figure 1:**
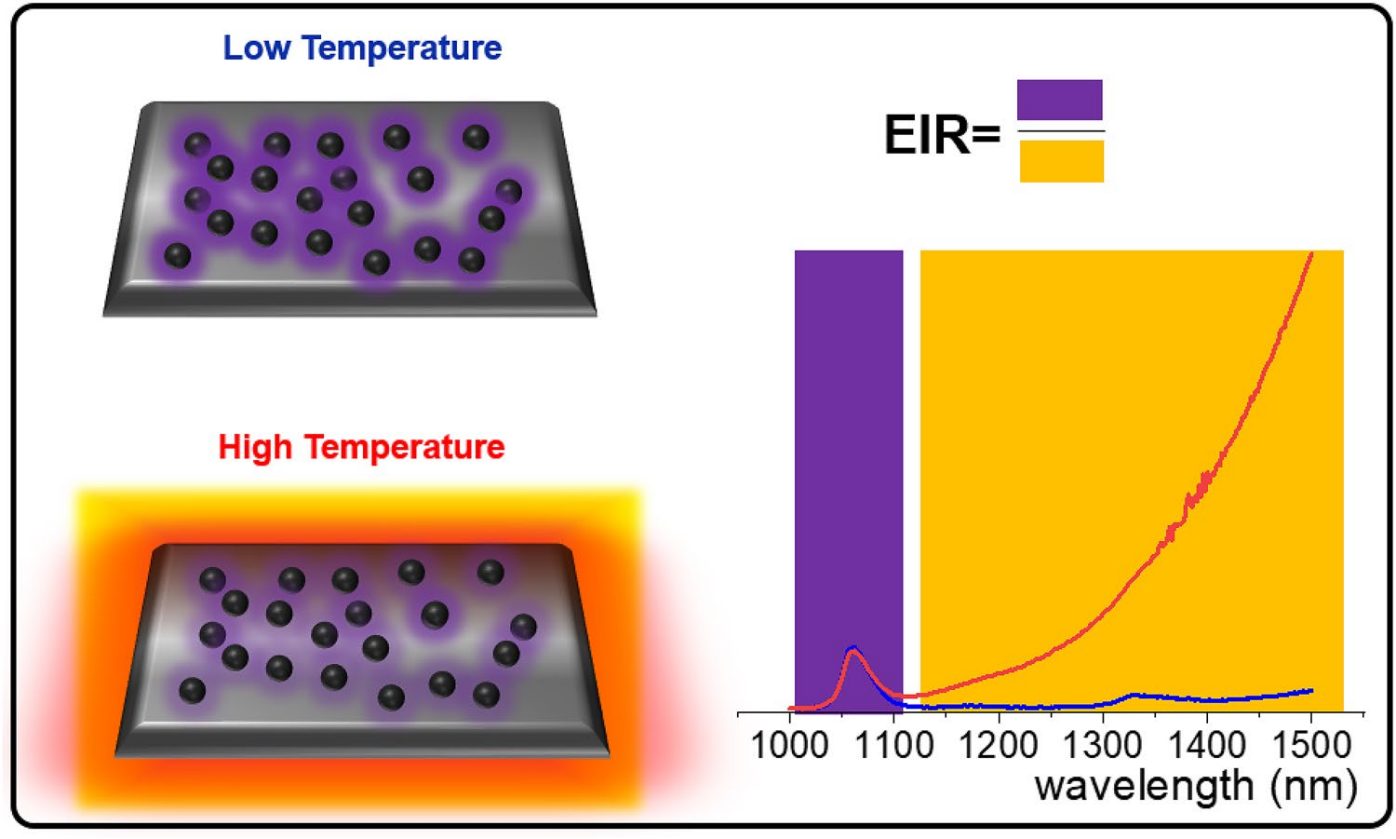
Concept of the emissive noncontact thermometer: at low temperatures strong NIR emission of Nd^3+^ doped glass can be observed and thermal emission intensity is negligible (blue spectrum). The increase of temperature decreases Nd^3+^ emission intensity and enhance thermal emission (red spectrum). Emission intensity ratio of emission originating from these two processes can be used as a ratiometric thermometric property—emission intensity ratio (EIR).

Doubtlessly, the temperature is one of the most important thermodynamical parameters which describes the rate and character of physical processes and chemical reactions^1–7^. Temperature is also an important diagnostic tool which informs about the presence of i.e. inflammations or cancerous cells presence for medical diagnosis or about the hot-spots or parts of mechanical elements exposed to increased risk of being damaged^4,8^. The fact, that most of the commercially available techniques of temperature readout operates in a contact mode, excludes the possibility to provide the information about temperature distribution in many fields, where contact mode may interfere with the analyzed system (biological or some electrical systems) or in some harsh environment (extreme temperatures or pH)^9^. Therefore, development of the noncontact temperature readout techniques is especially desired. One of the most widespread and most popular noncontact method of temperature determination exploits bolometric thermovision technique (thermovising cameras), which are taking advantage from the thermal infrared emission (TE) of the objects to visualize temperature distribution. The easiness and truly noncontact mode are one of the most important advantages of this technique. However, this method reveals some disadvantages associated with high costs, and its strong dependence of temperature determination accuracy on sample emissivity – i.e. the input parameter which in the most of the cases is hard to know or estimate. Additionally, thermovision camera provide information about the temperature of only the first object on the optical path or about the surface of the objects. Therefore, it cannot be applied for microscopy imaging or biomedical volumetric sub-tissue temperature distribution evaluation. Actually, as it was shown, temperature of the nanoparticles localized 2 mm below the surface of the skin was 8 °C lower, when read by the thermovision cameras compared to intra-volume luminescent thermometry based method^4,10–16^. The latter, is an interesting alternative for thermovision cameras—this technique takes advantage from the thermal changes of luminescent properties of the phosphor to determine the temperature of the phosphor and its surrounding. In this case the downsides of using thermovision cameras are eliminated. Moreover, luminescent thermometry provide information about real temperature of the object, therefore can be used in majority of biomedical applications. Due to low scattering and absorption of the light by the tissue, near infrared (NIR) spectral range, where optical transparency windows of tissues occur, is especially desirable to report temperature dependent spectral signatures. Up to date many different phosphors have been proposed as NIR emitting noncontact temperature sensors including: quantum dots^17–19^, silicon nanoparticles^20^, nanogels^21^, inorganic nanoparticles doped with lanthanide ions^22–28^ or transition metal ions^29–34^, nanodiamonds^35^, metal–organic frameworks^36^, coordination polymers^37^, semiconductor nanoparticles^38^ etc. The progress in luminescence based thermometry, is strongly related to the advancement in available materials, especially because low intensity NIR emission of the phosphors inevitably increases the uncertainty of the temperature determination. Moreover, because of thermal quenching of the phosphor luminescence, the accuracy of temperature determination decreases with increasing temperature. Thermal radiation has been used for temperature determination by several research groups^1,9^. However, most of the studies presented up to date, utilize the fitting of the spectra tail of the thermal radiation curve using Plack’s equation^1,9^. The obtained results gain accuracy proportionally to the spectral range used for fitting, which in general requires hyperspectral wide range NIR/MIR spectrometers. Therefore as it will be shown here taking advantage from the opposite thermal monotonicity of thermal radiation and Ln^3+^ luminescence the simplification of the experimental setup and enhancement of the sensitivity of temperature sensor to temperature changes are achieved. Therefore, in this communication we propose the combination of Nd^3+^ NIR luminescence and glass thermal emission toward the development of highly sensitive, ratiometric emissive noncontact thermometer. Such approach gains from the opposite thermal dependence of Ln^3+^ luminescence and spontaneous thermal emission of the objects. Oxyfluorotellurite (TZPN) glass doped with Nd^3+^ ions was used as a study case. This type of glass material merges good thermal and chemical stability of oxides with low phonon energy, high refractive index and broad optical transmittance region characteristic for fluorides. Consequently, TZPN glass material is a reasonable compromise between the pure oxide and fluoride glass^39,40^. In order to develop the NIR operating ratiometric emissive thermometer, lanthanide ions exhibiting intense NIR emission need to be selected. Additionally, temperature sensitivities that relate lanthanide emission to thermal emission will be maximized, when lanthanide emission is thermally quenched while thermal emission is enhanced in response to rising temperature. However, the rate of thermal quenching of Ln^3+^ ions must not be too fast since otherwise, temperature operating range of this ratiometric emissive thermometer can be too narrow. These desirable features made us select Nd^3+^ emitters, which is well known intense emission band at 1060 nm under c.a. 800 nm photoexitation The concept of our approach is explained in Fig. 1. At relatively low temperatures (below 180 °C), the intense NIR emission associated with the ^4^F_3/2_ → ^4^I_11/2_ and ^4^F_3/2_ → ^4^I_13/2_ electronic transitions of Nd^3+^ can be observed at 1060 nm and 1325 nm, respectively (violet part of the spectrum). The intensity of those bands decreases via multiphonon relaxation of the Nd^3+^ excited states. On the other hand, the thermal emission of the glass matrix increases with temperature (orange part of the spectrum).

Therefore, by the integration of emission intensity in the two spectral ranges i.e. 1500 nm for TE and 1060 nm for Nd^3+^ a ratiometric luminescent thermometer can be created.

## Results and discussion

**Figure 2:**
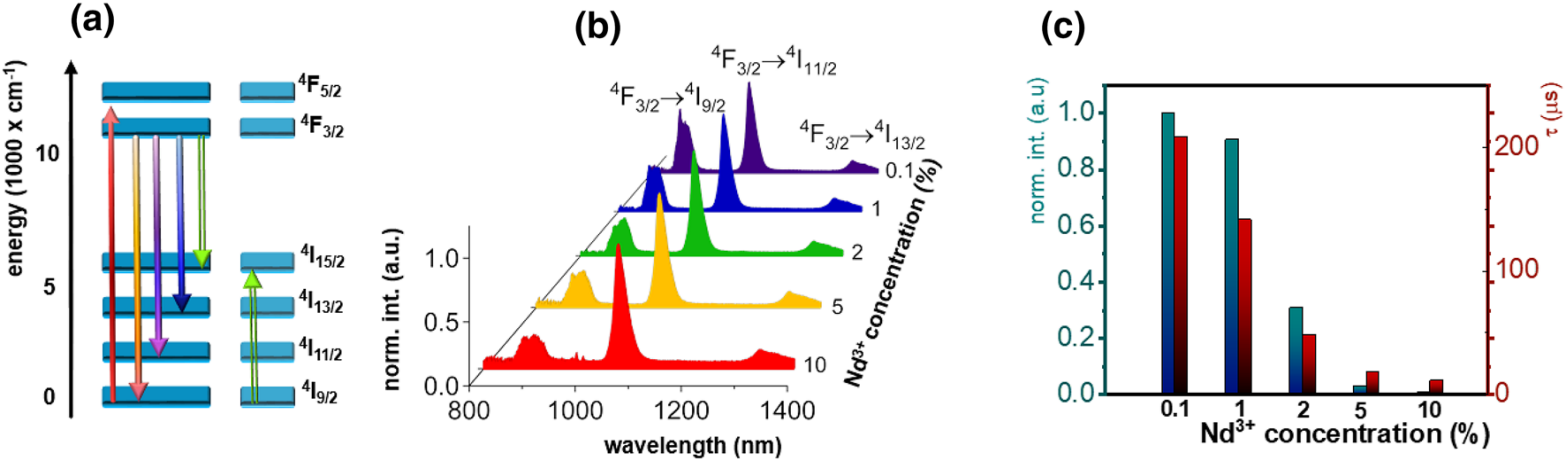
Simplified energy diagram of Nd^3+^ ions (**a**), the comparison of room temperature emission spectra of TZPN glasses for different Nd^3+^ concentration (normalized to the ^4^F_3/2_ → ^4^I_11/2_ emission band intensity) (**b**), the influence of Nd^3+^ concentration on the total emission intensity (blue bars) and lifetime (red bars) (**c**).

The luminescence of Nd^3+^ ions in TZPN glass upon λ_exc_ = 808 nm excitation can be explained by the use of simplified energy levels diagram of Nd^3+^ ions (Fig. 2a). The excitation, followed by the nonradiative depopulation processes leads to the population of emitting metastable ^4^F_3/2_ state. Its radiative depopulation causes the occurrence of three emission bands (Fig. 2b) at 880 nm, 1060 nm and 1350 nm attributed to the ^4^F_3/2_ → ^4^I_J_ (J = 9/2, 11/2 and 13/2, respectively) electronic transitions. However, when the dopant concentration increases, the interionic interactions, such as reabsorption and cross relaxations become dominant over luminescence^41^. First of them is responsible for the modification of the ^4^F_3/2_ → ^4^I_9/2_ emission band, while the later one changes the population of the ^4^F_3/2_ emitting state. This is manifested as decreasing the total integral emission intensity (Fig. 2c) and the shortening of the average luminescence lifetime from 210 µs for 0.1%Nd^3+^ to 11 µs for 10%Nd^3+^ ions (Fig. 2c, luminescence decay curves are presented in Figure S1.).

**Figure 3:**
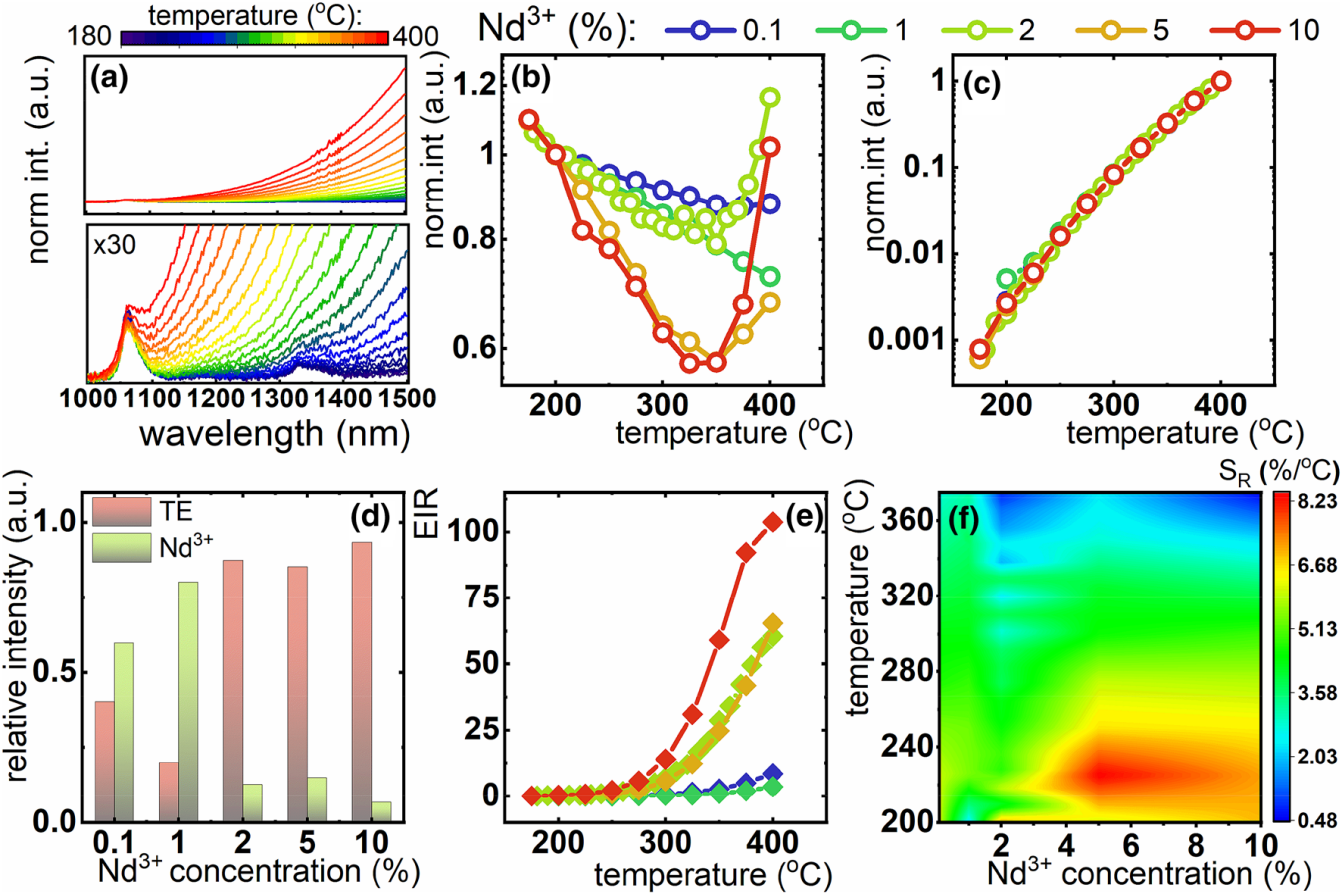
Representative emission spectra of TZPN glass doped with 2% of Nd^3+^ ions and its × 30 magnification (**a**), thermal evolution of integral emission intensity of Nd^3+^ luminescence (**b**) and of thermal emission (**c**) for different Nd^3+^ concentration; the contribution of Nd^3+^ and thermal emission to the total emission intensity at 300 °C for different Nd^3+^ concentration; thermal evolution of EIR (zoomed < 250 °C temperature range can be found in Fig. S9) (**e**) and S_R_ (**f**) for different Nd^3+^ concentration.

The increase of the temperature leads to thermal quenching of the Ln^3+^ emission intensity. In the case of Nd^3+^ ions this effect is caused by two thermally activated processes: (i) multiphonon depopulation of the ^4^F_3/2_ state and (ii) thermally activated cross relaxation process^42^. Therefore, reduction of the Nd^3+^ emission intensity is expected at elevated temperatures. On the other hand the thermal emission is governed by the Planck law of the black body emission:

1 $$ I\left( {\lambda ,T} \right) = A\frac{{hc^{2} }}{{\lambda^{5} \left( {\exp \left( {\frac{hc}{{\lambda kT}}} \right) - 1} \right)}} $$

where h, c, λ, k and A are Planck constant, speed of light, Boltzmann constant and emissivity of the object. As expected, the intensity of thermal emission increases with temperature. The representative thermal evolution of emission spectrum of TZPN:Nd^3+^ doped glass is presented in Fig. 3a). At temperatures around 100 °C two emission bands at 1050 nm and 1350 nm corresponding to ^4^F_3/2_ → ^4^I_11/2_, ^4^F_3/2_ → ^4^I_13/2_ electronic transitions of Nd^3+^ ions can be clearly observed. On the other hand, as temperature increases, the thermal emission gradually increases. Already above 190 °C, the TR emission dominated the one from Nd^3+^ ions. According to the Planck’s law, the thermal emission undergoes a blue shift with an increase of temperature. Therefore, it spectrally overlaps with ^4^F_3/2_ → ^4^I_11/2_ emission band above 320 °C which decreases the temperature determination accuracy at higher temperatures and requires careful background correction for reliable and quantitate analysis. The thermal evolution of Nd^3+^ emission intensity for different Nd^3+^ concentrations reveals that for higher Nd^3+^ concentration the emission intensity is more strongly affected by temperature. For 0.1%Nd^3+^ the integral emission intensity decreases by 10% in the 150–350 °C temperature range, while for 10% of Nd^3+^ its intensity decreases by 40% (Fig. 3b). On the other hand, the thermal emission increases by three orders of magnitude in the 150–400 °C temperature range. As expected its thermal evolution is independent on the Nd^3+^ concentration (Fig. 3c).

Actually, emission spectra measured for the TZPN: Nd^3+^ glasses without any photoexcitation, confirms that the observed thermal emission in not associated with the light-to-heat conversion of the light absorbed by the Nd^3+^ ions but is an immanent thermal emission of the glass (Fig. S2). Almost perfect agreement between externally induced increased sample temperature and the temperature obtained from the fitting of the spectrum tail to thermal emission behavior (Eq. 1) was found, which is an additional confirmation that the observed emission is induced by the temperature rise (Fig. S3). The opposite monotonic behavior of thermal dependences originating from Nd^3+^ luminescence and from thermal emission suggests, that their integral emission intensity ratio (EIR) should be exceptionally susceptible to temperature variation and thus may become a promising thermometric parameter:

2 $$ EIR = \frac{{\int_{1480nm}^{1500nm} {TE} }}{{\int\limits_{1050nm}^{1070nm} {{}^{4}F_{3/2} \to {}^{4}I_{11/2} \left( {Nd^{3 + } } \right)} }} $$

From thermometric perspective, the high Nd^3+^ concentration facilitates the noncontact temperature readout. However, luminescence concentration quenching which occurs at higher Nd^3+^ concentrations, affects the integral Nd^3+^ emission intensity (Fig. 3d), which in turn reduces the accuracy of temperature determination. The contribution of the Nd^3+^ emission to the total emission spectrum is lower in the case of TZPN:10%Nd^3+^ in respect to the counterpart doped with 5%Nd^3+^ (Fig. 3d). The emission intensity and hence the signal to noise ratio strongly affect the accuracy and reliability of temperature readout^43^. Therefore, although EIR increases by two orders of magnitude for 10% of Nd^3+^ ions, the TZPN: 5% Nd^3+^ glass seems to suit the requirements of noncontact temperature sensing better (Fig. 3e). The relative sensitivity was calculated as follows^44^:

3 $$ S_{R} = \frac{1}{EIR}\frac{\Delta EIR}{{\Delta T}} \cdot 100\% $$

where ΔEIR represents the change of EIR corresponding to the change of temperature ΔT.. The S_R_ enables quantitative evaluation of various materials (i.e. in response to Nd^3+^ concentration) in various temperature ranges (Fig. 3f). These results indicate that the highest relative sensitivity S_R_ = 8.2%/°C was found for 5% of Nd^3+^ at 220 °C. This relative sensitivity is significantly higher that the SR obtained for particular emission signal only (S_R_ = 4.5%/°C for TE and 0.25%/°C for Nd^3+^ luminescence, see Fig. S4) which confirms the advantage of the thermometric performance of the EIR approach. Moreover, the use of NIR part of the thermal emission band, in contrary to mid-NIR spectral range used by thermovision camera, enables to directly monitor temperature of objects. Thermovision camera exploits 7–14 μm wavelength range for which most of the objects are not transparent. In the analyzed temperature range the cooling rate is very fast, therefore hysteresis of thermal change of EIR value was not observed (see. Fig. S5a) and this kind of noncontact temperature sensor reveals perfect stability which was confirmed within 30 heating–cooling cycles (Fig. S5b)^45^. After the statistical analysis of obtained that it was found that the discrepancy in EIR determination was below 1% of its value. The temporal stability of EIR readout was confirmed during 30 min-long experiment at 350 °C for TZPN:5%Nd^3+^ (Fig. S6). The suitability of TZPN:5%Nd^3+^ glass in EIR scheme for practical applications is further confirmed by the relatively low temperature determination uncertainty which is below 1% up to 280 °C and below 2 °C up to 400 °C (Fig. S7). Besides the high relative sensitivity to temperature changes, one of the most important advantage of EIR approach is the fact that thermal emission has a positive thermal coefficient, i.e. the intensity rises as the temperature rises. This is in contrary to the majority of the known luminescent thermometers. Additionally, in the case of the remote temperature readout, due to the dispersive dependence of the extinction of water molecules, the e.g. atmospheric humidity may affect the reliability of temperature readout^46^ (see Fig. S8). However, the performed analysis (see. Eq. S2–S6), reveals that when the extinction coefficient at particular emission wavelengths are known, the EIR-based temperature readout can be performed independently on the object-to-detector distance or the variable humidity. However, the disadvantage is the fact that the usable temperature range is rather limited due to the spectral overlap between lanthanide emission and thermal radtaion occurred at higher temperatures and the fact that EIR is more suitable to implement at higher temperatures at which thermal emission can be observed in NIR spectral region.

## Conclusions

A series of oxyfluorotellurite (TZPN) glasses doped with Nd^3+^ ions with different concentrations in the range of 0.1—10% was prepared. Spectroscopic measurements of the TZPN glasses were made under 808 nm excitation in a wide temperature range from 150° C to 400° C. In the 1000–1700 nm spectra range, two emission bands at 1060 nm and 1325 nm associated with ^4^F_3/2_ → ^4^I_11/2_ and ^4^I_13/2_ transitions, respectively, were observed, whose intensity decreased with increasing temperature.

At temperatures above 180 °C the appearance of a wide emission band at around 1480 nm was also noted. The intensity of this band increased with temperature and its maximum was blue-shifting at elevated temperatures. This band was attributed to thermal emission, whose origin was confirmed in an independent experiment without optical excitation, where externally delivered thermal energy gave rise to the observed emission. The results were in almost perfect agreement with fitting curves associated with Planck law of the black body emission. The impact of Nd^3+^ ion concentration was also examined. A decrease in the luminescence intensity of Nd^3+^ ions along with the increase in their concentration and the lack of concentration relationship for thermal emission of the matrix was noticed. Due to the opposite trend of changes of the intensity of Nd^3+^ luminescence and thermal emission of glasses, their ratio served as temperature-dependent EIR parameter. Unprecedently high relative sensitivity S_R_ = 8.2%/°C was obtained for 5% of Nd^3+^ at 220 °C. The proposed ratiometric approach, which involves thermal radiation and lanthanide luminescence, demonstrates a few important conceptual novelties with respect to the thermometers that involves fitting of the tail of thermal emission using Planck’s equation. These innovations aim to (1) simplify the technical means and instrumentation required for T determination by studying emission intensities at two emission bands, (2) improve the sensitivity by the opposite thermal behavior of thermal emission and the luminescence, and (3) eliminate or diminish the issues related to the possible impact the sample may have on accuracy of temperature reading by narrowing the focus to two distinct and well defined wavelength ranges. These results confirm that the analyzed emission intensity ratio (EIR) is extremely susceptible to temperature changes.

## Methods

The oxyfluorotellurite (TZPN) glass samples were fabricated from a mixture of high purity (4 N or 5 N, Alfa Aesar) powders of tellurium oxide (TeO_2_), anhydrous zinc fluoride (ZnF_2_), lead oxide (PbO), niobium oxide (Nb_2_O_5_) and neodymium oxide (Nd_2_O_3_). The obtained glasses revealed the following chemical compositions: (in mol%) (65-x)TeO_2_-20ZnF_2_-12PbO-3Nb_2_O_5_-xNd_2_O_3_ (x = 0.1, 1, 2, 5 and 10). Mixed in a dry box 10 g batch of the reagents were placed in corundum crucible and then melted in a resistance furnace at 830 °C for 30 min in normal atmosphere. The melt was poured onto a preheated brass plate and then was annealed for four hours below the glass transition temperature 360 °C in order to eliminate internal stresses. It was found that glass transition temperature T_g_ is constant independently on Nd^3+^ concentration in glasses. Contrary to this finding, initiation temperatures of crystallization T_c_ are slightly dependent on Nd_2_O_3_ concentrations used for the synthesis. The TZPN glass used in this study were plates of 10 mm in diameter and 3 mm of thinness. The values of density and refractive index were equal d = 5.76 g cm^−3^ and n = 1.98, respectively^40^. More detailed characterization of these glass materials can be found here^39,40^.

The emission spectra as well as luminescence decay profiles were measured using FLS980 Fluorescence Spectrometer from Edinburgh Instruments. Both the excitation and emission 300 mm focal length monochromators were in Czerny Turner configuration. Emission arm was supplied with ruled grating, 1800 lines/mm blazed at 500 nm. The 450 W Xe lamp was used as an excitation source. The spectral resolution was 0.1 nm. A R5509-72 photomultiplier tube from Hamamatsu in a nitrogen-flow cooled housing was used as a detector. Spectra were collected with the 0.5 spectral resolution and 0.1 s integrating time at each point. The temperature of the sample was controlled using a THMS 600 heating stage from Linkam (0.1 °C temperature stability and 0.1 °C set point resolution). Measurements were performed with the 1 min interval between measurements in order to obtain the thermal equilibrium.

The average lifetime of the ^4^F_3/2_ state of Nd^3+^ ions was calculated as follows:

4 $$ \left\langle \tau \right\rangle = \frac{{\int {I\left( t \right)tdt} }}{{\int {I\left( t \right)dt} }} $$

## Supplementary information

Supplementary Information
