# Supplementary material for: Synergy between NIR luminescence and thermal emission toward highly sensitive NIR operating emissive thermometry
Source: Sci Rep. 2020 Nov 12;10:19692. doi: 10.1038/s41598-020-76851-3 (PMC7664999; doi:10.1038/s41598-020-76851-3)
Supplement: Supplementary file 1 — Supplementary Information [file 41598_2020_76851_MOESM1_ESM.docx]

Supporting information

Synergy between NIR Luminescence and thermal emission toward highly sensitive NIR operating emissive thermometry

Lukasz Marciniak,* Karolina Trejgis , Radosław Lisiecki and Artur Bednarkiewicz

Włodzimierz Trzebiatowski Institute of Low Temperature and Structure Research, Poland

Corresponding author: [l.marciniak@intibs.pl](mailto:l.marciniak@intibs.pl)


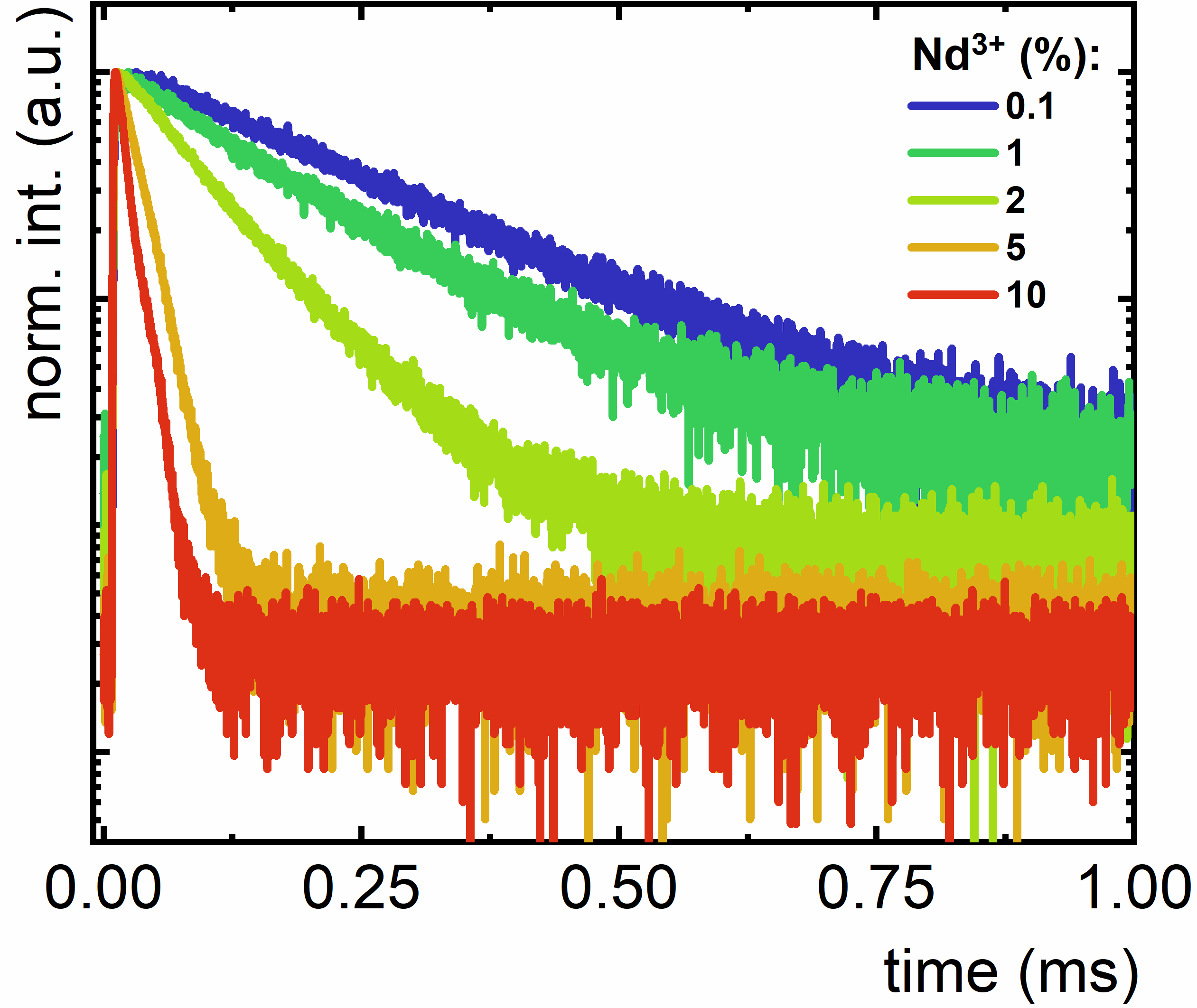


Figure S1. The luminescence decay profiles of ^4^F_3/2_ (λ_exc_=1060 nm, ^4^F_3/2_→^4^I_11/2_) state of Nd^3+^ ions in TZPN:Nd^3+^ glass with different Nd^3+^ concentration measured upon λ_exc_=808 nm.

**

**

Figure S2. The emission spectra of TZPN: Nd^3+^ glass with the fitted with the Planck curve using equation 1 measured as a function of temperature upon lamp excitation.


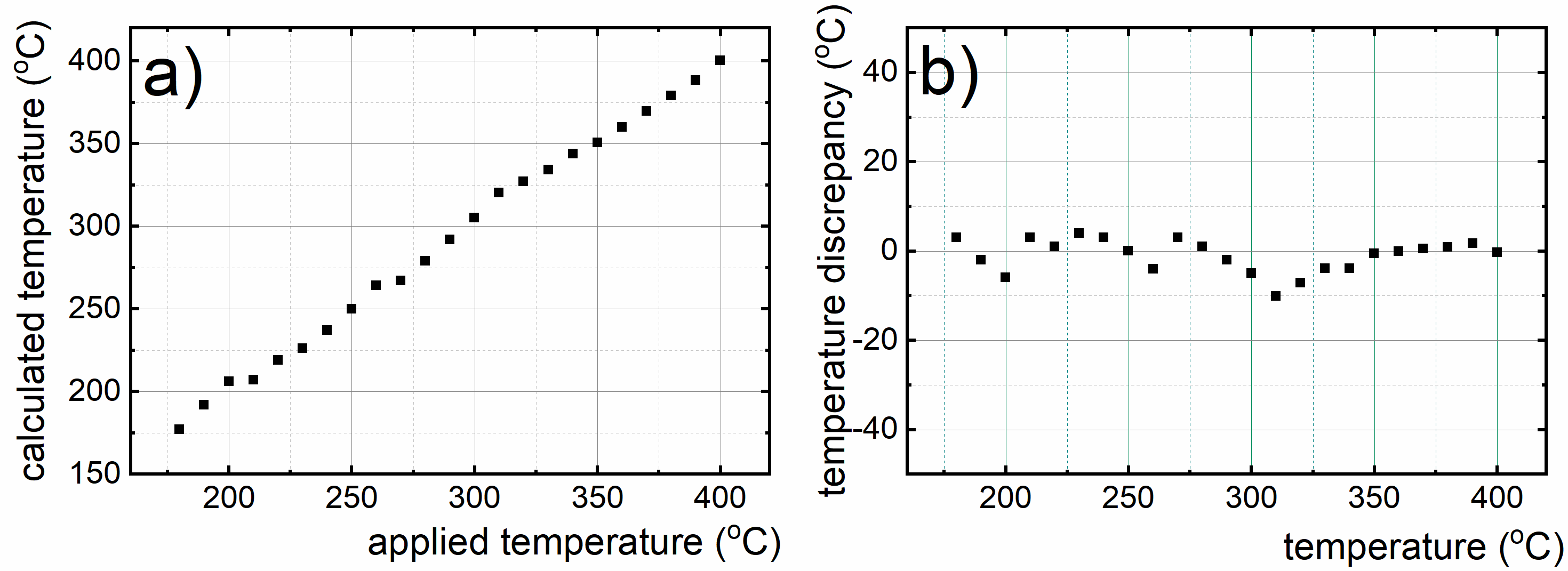


Figure S3. The calculated glass temperature using equation 1 as a function of applied temperature a) and the discrepancy between calculated and applied temperatures b)


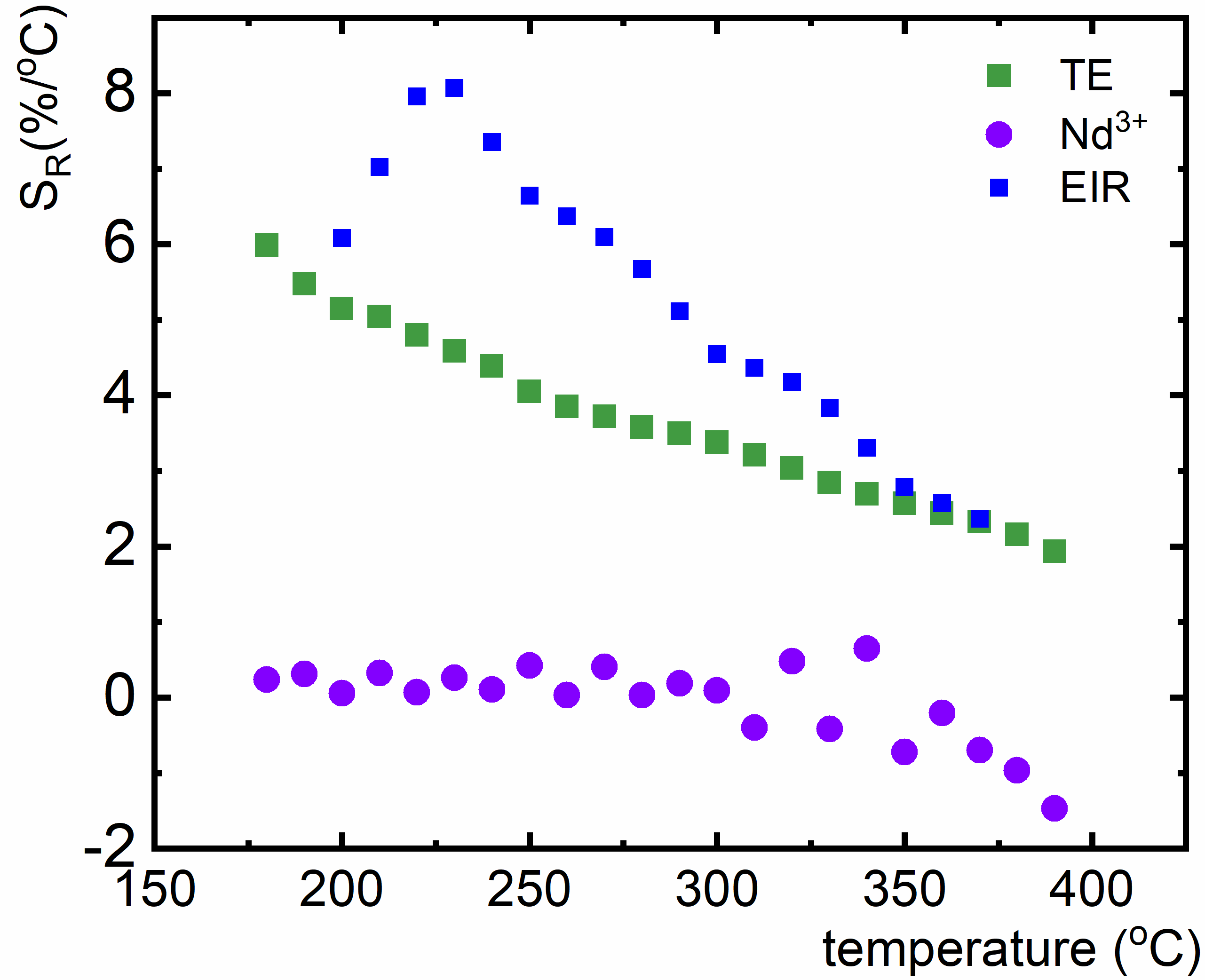


Figure S4. Comparison of the relative sensitivities for TZPN:2%Nd^3+^ glass using TE only, Nd^3+^ luminescence only and the EIR approach


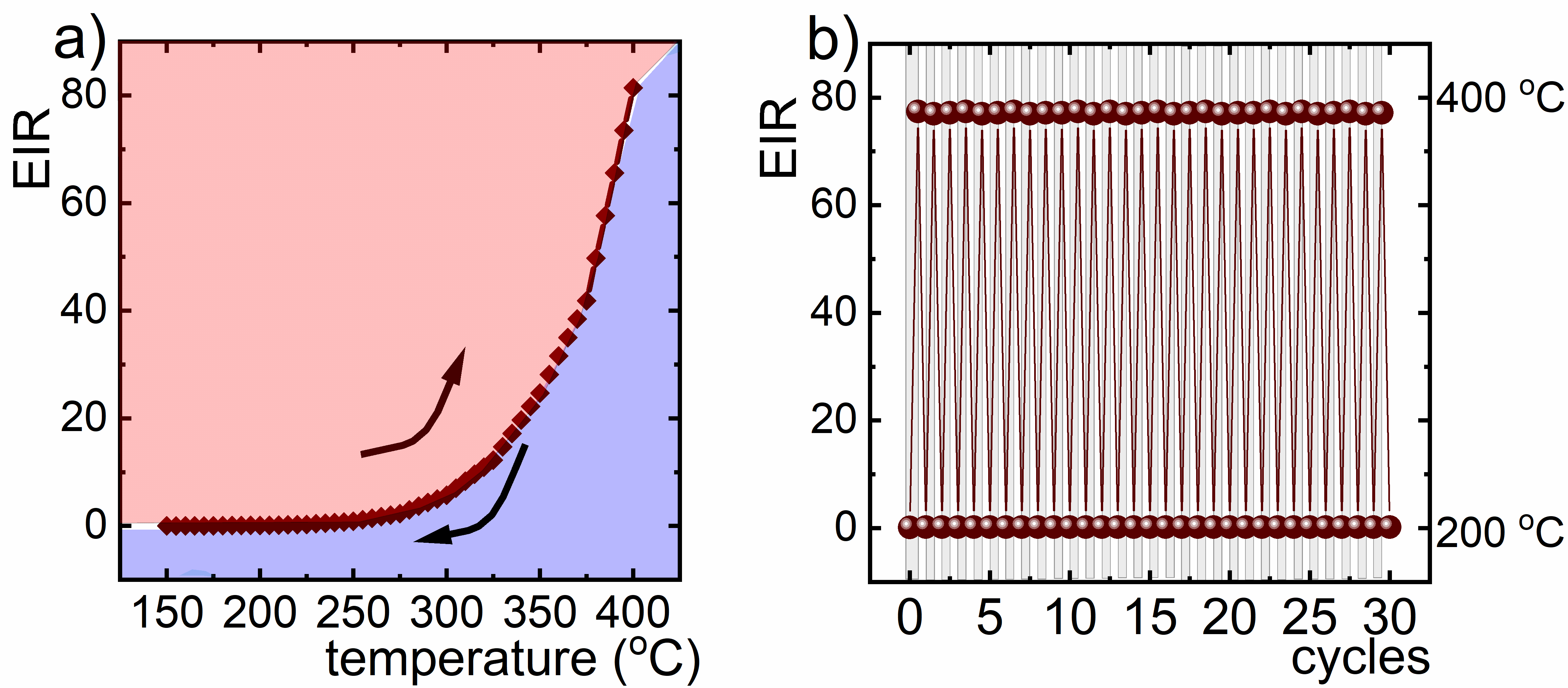


Figure S5. The EIR as a function of temperature obtained within increase and decrease (measured every 5^o^C) of temperature a) and the repeatability of the EIR readout within 30 heating cooling cycles b).


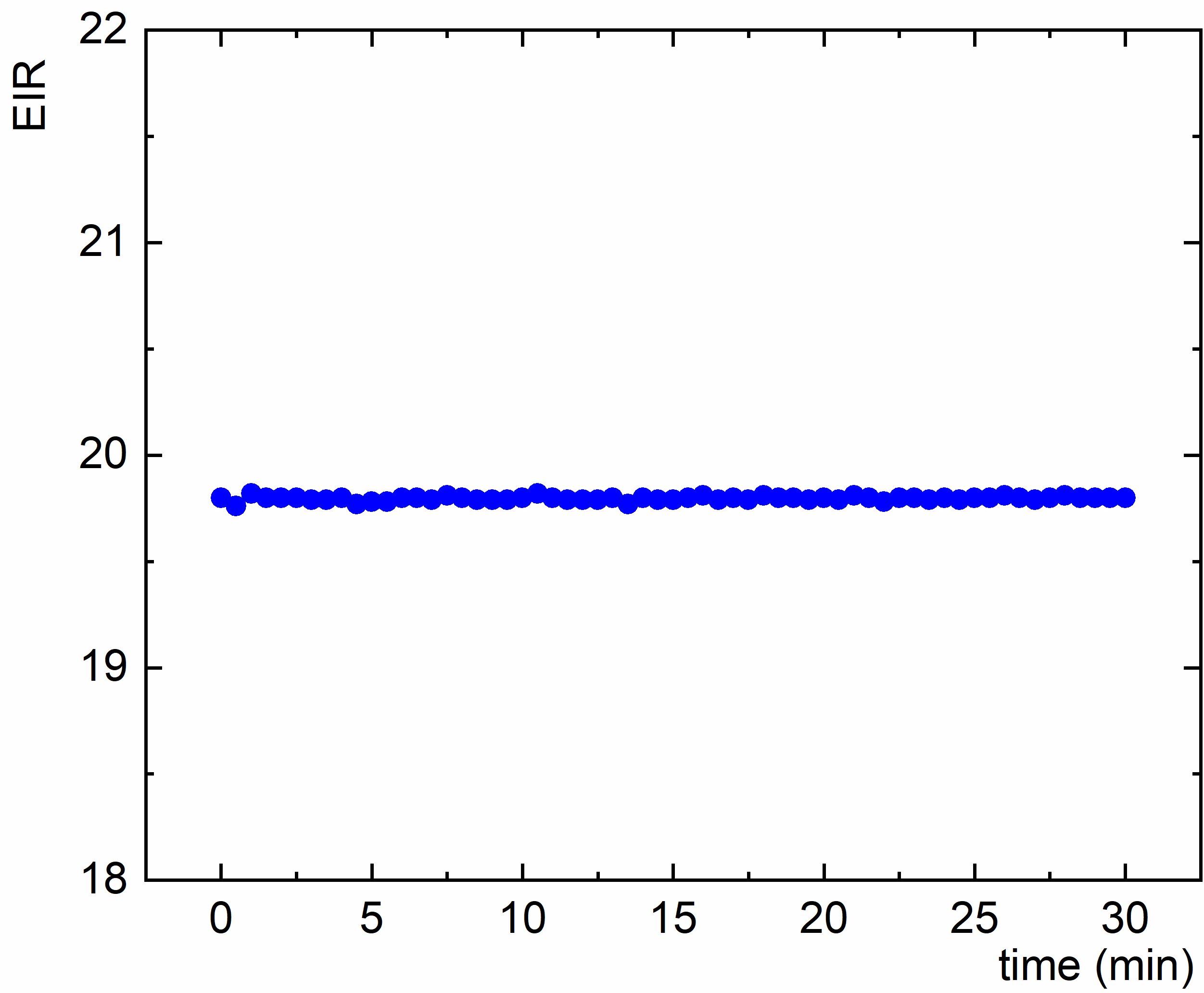


Figure S6. The EIR measured at 350^o^C as a function of time for TZPN:5%Nd^3+^ glass.

Temperature determination uncertainty was calculated using eq.S1:

$\delta T=\frac{1}{S_{R}}\frac{\delta EIR}{EIR}$ (eq. S1)

S_R_ is the relative sensitivity and δEIR/EIR determines the uncertainty of the EIR determination.


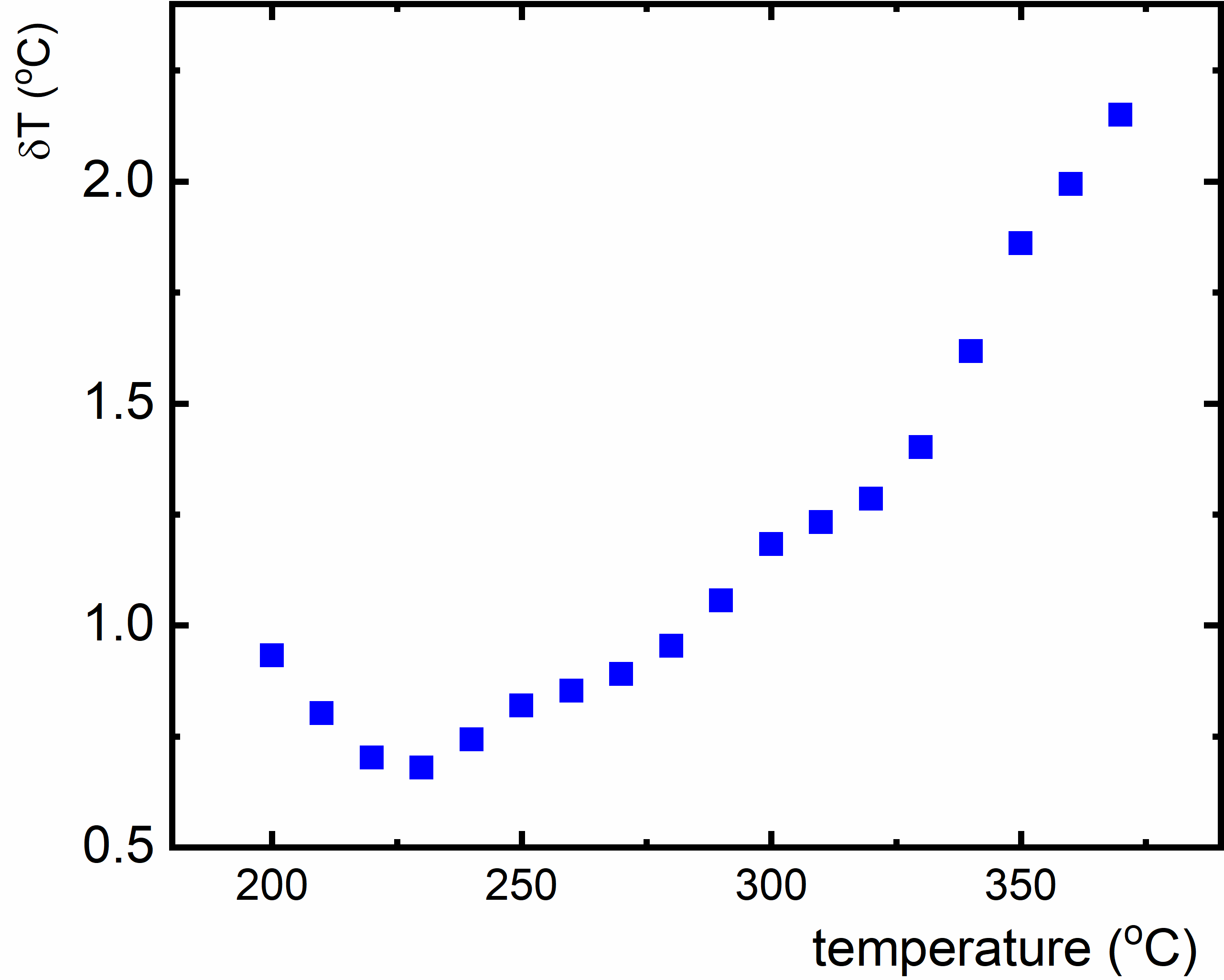


Figure S7. The uncertainty of temperature determination using EIR approach in TZPN:2%Nd^3+^

*The humidity effect on the reliability of temperature readout basing on EIR-approach*

The emission from T sensor (composed of lanthanide emission and thermal emission) being watched from a distance through a medium exhibiting various humidity, will give different variable response. Following Labeert-Beer law

${log}_{10}\frac{I_{0}}{I}=\varepsilon CL$ Eq.S2

where L is the distance between emissive thermometer and the detection system, I_0_, I, represent emission intensity at L=0 and L, respectively, C is the water molecules concentration and ԑ_Nd3+_ is the light extinction at given emission wavelength. One needs to know both the humidity (proportional to C), extinction coefficient at given wavelength (1060 for lanthanide or 1500 nm for TE), and the path length between the T sensor and the detector. Because the thermal emission is broad, one can select two spectral parts of band (let’s call is X and Y) from thermo-emission spectral range. Thus

$\frac{I_{X}}{I_{Y}}=\frac{I_{Xo}\cdot e^{-\varepsilon_{X}CL}}{I_{Yo}\cdot e^{-\varepsilon_{Y}CL}}={EIR}_{o}^{'}$ Eq.S3

When the integral intensities ratio between X and Y is known at the sample $\left( {EIR}_{o}^{'}=\frac{I_{Xo}}{I_{Yo}} \right)$ and at arbitrary distance $({EIR}^{'}=\frac{I_{X}}{I_{Y}})$ are known at the same temperature the unknown cL factor may be calculated:

$cL=ln\left[ \frac{I_{X}}{I_{Y}}\cdot\frac{I_{Yo}}{I_{Xo}} \right]^{\varepsilon_{X}-\varepsilon_{Y}}$ Eq.S4

Because the ratio between intensities at emission bands X and Y obviously depends of the humidity and path length, but do not depend on temperature, this enables to correct the actual ERI calibration curve measured at the sample (${EIR}_{o}(T)$) to a new calibration curve ($EIR(T)$) at arbitrary distance from the sensor with unknown humidity of environment:

$\frac{I_{Nd}(T)}{I_{Y}(T)}=\frac{I_{Ndo}(T)\cdot e^{-\varepsilon_{Nd}CL}}{I_{Yo}(T)\cdot e^{-\varepsilon_{Y}CL}}$ Eq.S5

Thus

$EIR(T)={EIR}_{o}(T)\cdot e^{-cL(\varepsilon_{Nd}-\varepsilon_{Y})}$ Eq.S6

The temperature may be easily determined from combined Eq.5 and Eq.3. To make it happen, one needs to (1) know the calibration curves of pristine thermometer (${EIR}_{o}\left( T \right)$ and ${EIR}_{o}^{'}$ at known temperature T_C_, (2) at the same T_C_ temperature measure emission ${EIR}^{'}$ at given position of the detector against the sensor. The latter measurement will enable to determine CL (Eq.3), to let correct the whole ${EIR}_{o}(T)$ calibration curve to a new one $EIR(T)$, and from this, get actual temperature of the sample remotely, independently from the distance or the humidity


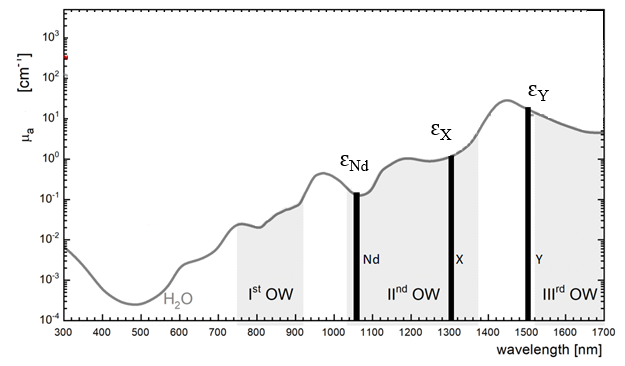


Figure S8. The dispersive dependence of the light extinction by water molecules


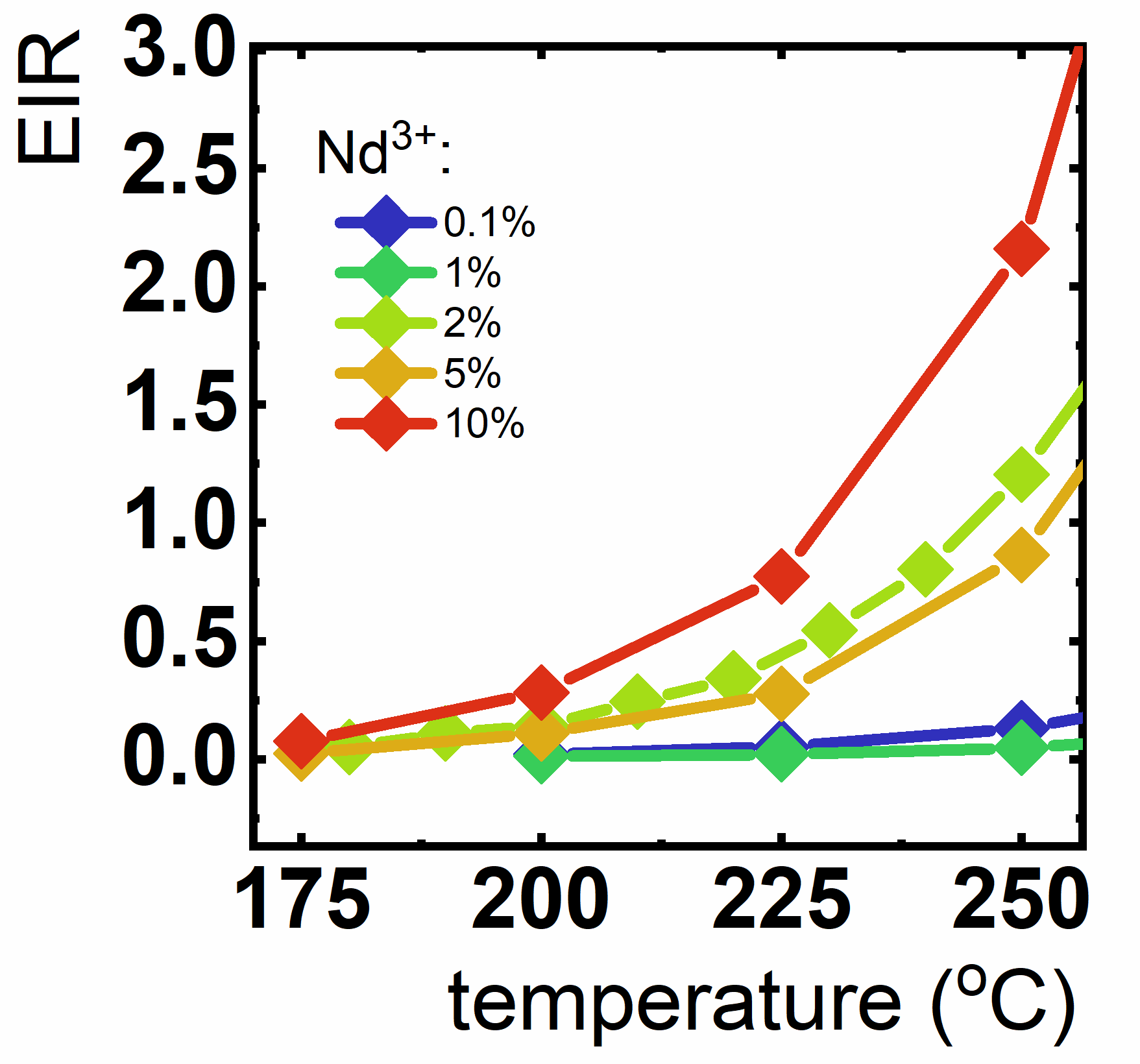


Figure S9. The thermal evolution (<250^o^C temperature range) of EIR in TZPN:Nd^3+^ for different Nd^3+^ concentration.
